# MiR155-5p Inhibits Cell Migration and Oxidative Stress in Vascular Smooth Muscle Cells of Spontaneously Hypertensive Rats

**DOI:** 10.3390/antiox9030204

**Published:** 2020-03-01

**Authors:** Nan Wu, Chao Ye, Fen Zheng, Guo-Wei Wan, Lu-Lu Wu, Qi Chen, Yue-Hua Li, Yu-Ming Kang, Guo-Qing Zhu

**Affiliations:** 1 Key Laboratory of Targeted Intervention of Cardiovascular Disease, Collaborative Innovation Center of Translational Medicine for Cardiovascular Disease, and Department of Physiology, Nanjing Medical University, Nanjing 211166, China; nanwu@njmu.edu.cn (N.W.); chaoye@njmu.edu.cn (C.Y.); fenzh@njmu.edu.cn (F.Z.); wanguowei2020@163.com (G.-W.W.); luluuu@njmu.edu.cn (L.-L.W.); 2 Department of Pathophysiology, Nanjing Medical University, Nanjing 211166, China; qichen@njmu.edu.cn (Q.C.); yhli@njmu.edu.cn (Y.-H.L.); 3 Department of Physiology and Pathophysiology, Cardiovascular Research Center, Xi’an Jiaotong University School of Medicine, Xi’an 710061, China; ykang@mail.xjtu.edu.cn

**Keywords:** microRNA, vascular smooth muscle cells, hypertension, oxidative stress, inflammation

## Abstract

Migration of vascular smooth muscle cells (VSMCs) is essential for vascular reconstruction in hypertension and several vascular diseases. Our recent study showed that extracellular vesicles derived from vascular adventitial fibroblasts of normal rats inhibited VSMC proliferation by delivering miR155-5p to VSMCs. It is unknown whether miR155-5p inhibits cell migration and oxidative stress in VSMCs of spontaneously hypertensive rats (SHR) and in angiotensin II (Ang II)-treated VSMCs. The purpose of this study was to determine the role of miR155-5p in VSMC migration and its underlying mechanisms. Primary VSMCs were isolated from the aortic media of Wistar-Kyoto rats (WKY) and SHR. Wound healing assay and Boyden chamber assay were used to evaluate VSMC migration. A miR155-5p mimic inhibited, and a miR155-5p inhibitor promoted the migration of VSMC of SHR but had no significant effect on the migration of VSMC of WKY. The miR155-5p mimic inhibited angiotensin-converting enzyme (ACE) mRNA and protein expression in VSMCs. It also reduced superoxide anion production, NAD(P)H oxidase (NOX) activity, as well as NOX2, interleukin-1β (IL-1β), and tumor necrosis factor α (TNF-α) expression levels in VSMCs of SHR but not in VSMCs of WKY rats. Overexpression of miR155-5p inhibited VSMC migration and superoxide anion and IL-1β production in VSMCs of SHR but had no impact on exogenous Ang II-induced VSMC migration and on superoxide anion and IL-1β production in WKY rats and SHR. These results indicate that miR155-5p inhibits VSMC migration in SHR by suppressing ACE expression and its downstream production of Ang II, superoxide anion, and inflammatory factors. However, miR155-5p had no effects on exogenous Ang II-induced VSMC migration.

## 1. Introduction

Vascular smooth muscle cells (VSMCs) are majorly located within the tunica media of vessels. VSMC migration from arterial media to intima is an essential event in the pathogenesis of several vascular diseases including atherosclerosis, hypertension, restenosis, transplant vasculopathy, and arterial wall injury [1,2]. VSMC migration and proliferation are crucial for vascular remodeling in pulmonary hypertension [3]. VSMC migration is involved not only in hypertension-related maladaptation of vascular remodeling but also in the development and progress of hypertension [4]. 

Oxidative stress is originated by an imbalance of reactive oxygen species (ROS) production and antioxidant mechanisms [5,6]. ROS are involved in several intracellular signaling pathways as secondary messengers [7]. However, a problem occurs when ROS production exceeds the capacity of antioxidant mechanisms, which further causes cellular damage and inflammation [8]. Oxidative stress greatly contributes to the pathogenesis of many cardiovascular diseases by promoting vascular inflammation and VSMC proliferation, migration, and apoptosis [9,10,11]. Vascular oxidative stress and inflammation are crucial for vascular remodeling in hypertension [12,13,14].

MicroRNAs (miRNAs) represent a class of small, 18- to 28-nuclotide-long, non-coding RNA molecules involved in the regulation of gene expression. They bind to the 3′-untranslated regions of their target mRNA sequence to negatively regulate its expression by inhibiting its translation or promoting its degradation [15]. It has been found that silencing of miR155 in human brain microvessel endothelial cells promotes cell proliferation and migration [16]. Recent studies in our lab have shown that miR155-5p level was reduced and angiotensin-converting enzyme (ACE) expression was increased in the aortic media of spontaneously hypertensive rats (SHR) compared with Wistar–Kyoto (WKY) rats. Extracellular vesicles derived from vascular adventitial fibroblasts of WKY inhibited the proliferation of VSMC of SHR by delivering miR155-5p to VSMCs [17]. Vascular adventitial fibroblast-derived extracellular vesicles in SHR promoted VSMC migration by conveying ACE to VSMCs [18]. However, the roles of miR155-5p in VSMC migration, oxidative stress, and inflammation are still not known. The present study was designed to investigate the roles and mechanisms of action of miR155-5p in cell migration, oxidative stress, and inflammation in VSMCs of SHR.

## 2. Materials and Methods

### 2.1. Animals

Male WKY and SHR aged 12 weeks were obtained from Vital River Lab Animal Technology Co. Ltd. (Beijing, China). The rats were caged in a temperature-controlled and humidity-controlled room with an alternating 12 h light-dark cycle and had free access to tap water and standard chow. The experimental procedures were approved by the Experimental Animal Care and Use Committee at Nanjing Medical University (No. 1811017) and were in accordance with the Guide for the Care and Use of Laboratory Animal (NIH publication, 8th edition, 2011). The systolic blood pressure in all the SHR rats used in this study was higher than 150 mmHg, meeting the criterion of hypertension.

### 2.2. Culture of Primary VSMCs

SHR is the best animal model of human essential hypertension. Angiotensin II (Ang II) greatly contributes to hypertension and vascular remodeling. Both VSMCs from SHR and Ang II-treated VSMCs from normal rats are widely used as in vitro models for the investigation of VSMC migration in hypertension. VSMCs were isolated and prepared from the media of the thoracic aorta of WKY and SHR, as we described previously [19]. Briefly, the perivascular adipose tissues and adventitia of the thoracic aorta were removed, and the aorta was cut open and stripped of the intima. The tissues were treated with Type 1A collagenase for digestion and then centrifuged for isolation of the cells. The isolated VSMCs were cultured in DMEM containing 10% fetal bovine serum (FBS), penicillin (100 IU/mL), and streptomycin (10 mg/mL) (Gibco, Grand Island, NY, USA) at 37 °C in an incubator with 5% CO_2_. VSMCs were identified by their substantial α-SMA expression (a marker of VSMCs) without detectable vimentin (a marker of fibroblasts) and PECAM-1 (a marker of endothelial cells) expressions. VSMCs from the second to the sixth passage were used for the experiments.

### 2.3. Boyden Chamber Assay

The Boyden chamber assay was used to evaluate VSMC migration and invasion, as we reported previously [20]. VSMCs were seeded into FBS-free medium in the upper chamber of a 24-well Transwell with 8 μm pore size (Merck kGaA, Darmstadt, Germany). Medium with 10% FBS was added into the lower chamber. The assay was allowed to proceed for 24 h. A cotton swab was used to scrape the cells from the upper surface of each filter. The cells that had moved to the lower surface of the membrane were stained with crystal violet. Stained cells were counted in five randomly selected fields. 

### 2.4. Wound-Healing Assay

A wound-healing assay was also used to examine VSMC migration, as we reported previously [20]. The isolated cells were seeded into a 6-well plate. A standard 1 mL pipette tip was used to scratch the near-confluent cells in the plate to form a gap. The cellular debris and floating cells were washed with PBS, fresh medium was added, and the cells were incubated for 24 h. The images of cell migration across the wound were taken at 0 h and 24 h with an inverted microscope (Axio Vert. A1, Zeiss, Oberkochen, Germany). The average migrated distance was calculated.

### 2.5. Transfection of miR155-5p Mimic and Inhibitor

The miR155-5p mimic, miR155-5p inhibitor, negative control (NC), and RNAifectin™ transfection reagent were obtained from Applied Biological Materials Inc. (Richmond, BC, Canada). After VSMCs were cultured for 16 h in 6-well plates (about 5 × 10^5^ cells per well), the cells were transfected with RNAifectin™ transfection reagent (6 μL) plus negative control, miR155-5p mimic (50 nmol/L), or miR155-5p inhibitor (100 nmol/L) for 6 h. Then, the culture medium was replaced to remove the transfection reagent. Measurements were performed 24 h after transfection.

### 2.6. MiR155-5p Overexpression and Ang II Treatment in VSMCs

A commercial control adenovirus (Ctrl-Ad) and AdmiRa-rno-miR155-5p (Ad-miR155-5p) (Applied Biological Materials Inc., Richmond, BC, Canada) were used to determine the roles of miR155-5p. Ang II (Sigma, St. Louis, Mo, USA) was used to induce VSMC migration and served as another model of VSMC migration. VSMCs in 6-well plates at 70% confluence were transfected with Ctrl-Ad or Ad-miR155-5p (40 MOI in 1 mL per well), placed in an incubator for 24 h and then treated with PBS or Ang II (100 nM) for another 24 h. Measurements were carried out 48 h after the transduction of Ctrl-Ad or Ad-miR155-5p.

### 2.7. Western Blot Analysis

The samples were homogenized in lysis buffer. Protein concentration of the supernatants was determined using a BCA protein assay kit (Santa Cruz, CA, USA). Total proteins were separated by sodium dodecyl sulfate-polyacrylamide gel electrophoresis (SDS-PAGE) and blotted to a polyvinylidene fluoride (PVDF) membrane. The Enhanced Chemiluminescence Detection Kit was obtained from Thermo Fisher Scientific (Rockford, IL, USA) and was used to detect protein bands. Antibodies against ACE, NAD(P)H oxidase 2 (NOX2), NOX4, and tumor necrosis factor α (TNF-α) were purchased from Proteintech Group Inc (Rosemont, IL, USA). The antibody against interleukin-1β (IL-1β) was purchased from Abcam (Cambridge, MA, USA). The antibody against GAPDH was obtained from Cell Signaling Technology (Beverly, MA, USA).

### 2.8. Measurement of miR155-5p Level with qPCR

The extraction of total RNA was carried out by miRcute miRNA isolation kit. The extracted RNA was quantified using NanoDrop 2000 Spectrophotometer (Thermo-Fisher Scientific, Wilmington, DE, USA), and 1 μg of total RNA was reverse-transcribed to cDNA, using the miRcute Plus miRNA First-Strand cDNA kit. Then, miRNA-specific primers and MiRcute Plus miRNA qPCR kit were employed to detect the mature miRNAs using the StepOnePlus™ Real-Time PCR System (Applied Biosystems, Foster City, CA, USA). U6 small nuclear RNA was used for normalization as an internal control. All kits used for the measurement of miR155-5p were purchased from Tiangen Biotech Co. (Beijing, China).

### 2.9. Measurement of ACE mRNA Level with qPCR

RNA was extracted with Trizol reagent (Life Technologies, Gaithersburg, MD, USA). PrimeScript^®^ RT reagent kits (Takara, Otsu, Shiga, Japan) and the ABI PRISM 7500 sequence detection PCR system (Applied Biosystems, Foster City, CA, USA) were used for reverse transcriptase reactions. Quantitative data were normalized to GAPDH expression. Primer sequences used in this study are listed in the online-only Data Supplement (Table S1).

### 2.10. DHE Fluorescence Staining

Dihydroethidium (DHE) fluorescence staining in VSMCs was used to evaluate intracellular ROS production. The cells (3 × 10^5^ cells/mL) were incubated in 6-well plates with PBS containing 10 μM of DHE in a dark and humidified container at 37 °C for 30 min and then were washed three times with PBS. Fluorescence was examined with a fluorescence microscope (DP70, Olympus Optical, Tokyo, Japan) under excitation at 518 nm and emission at 605 nm.

### 2.11. Measurement of NAD(P)H Oxidase Activity

NAD(P)H oxidase activity was examined with the lucigenin-derived chemiluminescence method [21,22,23]. Dark-adapted lucigenin and NAD(P)H (100 μM) were added to induce photon emission. Light emission was measured with a luminometer (Model 20/20n, Turner, CA, USA) for 10 times in 10 min. The values were expressed as relative mean light unit (MLU)/min/mg of protein. The light emission value of the buffer containing lucigenin (5 μM) was used as background chemiluminescence.

### 2.12. Statistics and Data Analysis

All data were expressed as mean ± SE. One-way or two-way ANOVA followed by post-hoc Bonferroni test was used for statistical analysis. Differences with *p* < 0.05 were considered statistically significant.

## 3. Results

### 3.1. Effects of miR155-5p Mimic and Inhibitor on VSMC Migration

**Figure 1:**
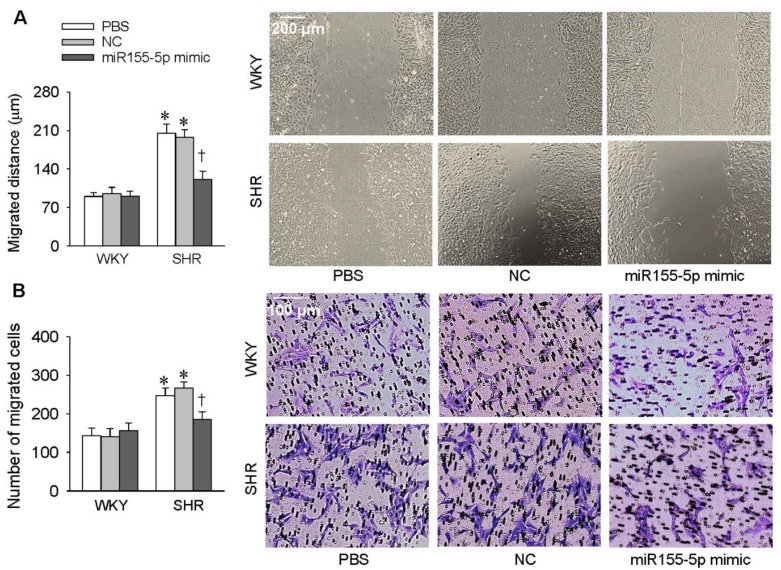
Effects of the miR155-5p mimic on vascular smooth muscle cells (VSMC) migration. VSMCs derived from Wistar-Kyoto (WKY) rats and spontaneously hypertensive rats (SHR) were treated with PBS, negative control (NC), or the miR155-5p mimic (50 nmol/L). Measurements were made 24 h after transfection. (**A**) VSMC migration evaluated by a wound healing assay. (**B**) VSMC migration evaluated by the Boyden chamber assay. Values are mean ± SE; * *p* < 0.05 vs. WKY; † *p* < 0.05 vs. PBS or NC; *n* = 6 per group.

**Figure 2:**
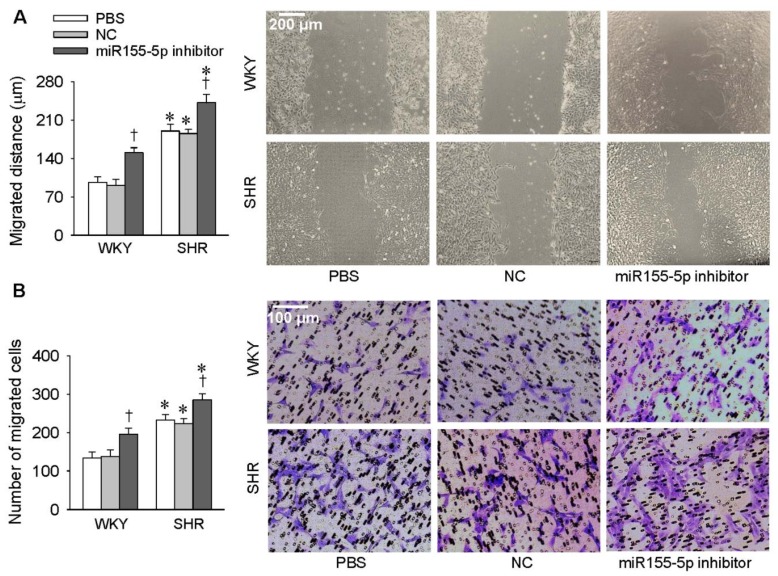
Effects of the miR155-5p inhibitor on VSMC migration. VSMCs from WKY rats and SHR were treated with PBS, negative control (NC), or the miR155-5p inhibitor (50 nmol/L). Measurements were made 24 h after transfection. (**A**) VSMC migration evaluated by a wound healing assay. (**B**) VSMC migration evaluated by the Boyden chamber assay. Values are mean ± SE; * *p* < 0.05 vs. WKY; † *p* < 0.05 vs. PBS or NC; *n* = 6 per group.

VSMC migration was evaluated with a wound healing assay and the Boyden chamber assay. Treatment of VSMC with the miR155-5p mimic attenuated the migration of VSMC derived from SHR but had no significant effect on VSMC from WKY rats (Figure 1A,B). Treatment with the miR155-5p inhibitor promoted the migration of VSMC from both WKY rats and SHR (Figure 2A,B). These results suggest that miR155-5p plays an important role in inhibiting the migration of VSMC from SHR.

### 3.2. Effects of miR155-5p Mimic and Inhibitor on ACE Expression

**Figure 3:**
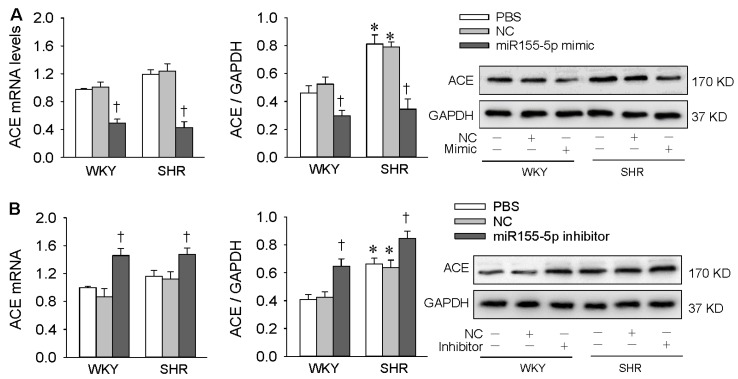
Effects of miR155-5p mimic and inhibitor on angiotensin-converting enzyme (ACE) mRNA and protein expression levels in VSMCs of WKY rats and SHR. VSMCs were treated with PBS, negative control (NC), miR155-5p mimic (50 nmol/L), or miR155-5p inhibitor (50 nmol/L. Measurements were made 24 h after transfection. (**A**) effects of miR155-5p mimic; (**B**) effects of miR155-5p inhibitor. Values are mean ± SE; * *p* < 0.05 vs. WKY; † *p* < 0.05 vs. PBS or NC; *n* = 4 per group.

MiR155-5p mimic inhibited ACE mRNA and protein expression in VSMCs of both WKY rats and SHR (Figure 3A), confirming our previous findings that ACE is one of the targets of miR155-5p, and miR155-5p negatively regulates ACE expression in VSMCs in rat [17]. The miR155-5p inhibitor increased ACE expressions in VSMCs of both WKY rats and SHR (Figure 3B), suggesting endogenous miR155-5p has a role in inhibiting ACE expression in WKY rats and SHR. It is well known that ACE promotes the conversion of Ang I to Ang II, and the latter promotes oxidative stress [24], inflammation [25], and VSMC migration [26]. It would be interesting to know whether miR155-5p could attenuate oxidative stress and inflammation in VSMCs of SHR.

### 3.3. Effects of miR155-5p Mimic on Oxidative Stress

**Figure 4:**
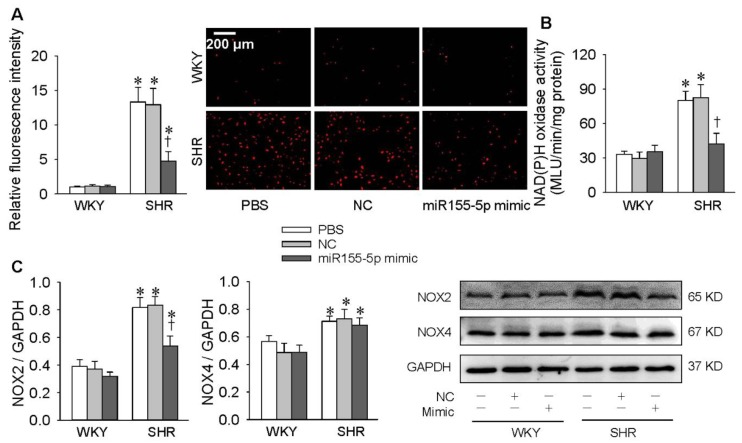
Effects of the miR155-5p mimic on oxidative stress in VSMCs of WKY rats and SHR. VSMCs were treated with PBS, negative control (NC), or the miR155-5p mimic (50 nmol/L). Measurements were made 24 h after transfection. (**A**) Dihydroethidium (DHE reactive oxygen species (ROS) in VSMCs. (**B**) NAD(P)H oxidase activity. (**C**) NOX2 and NOX4 protein expression levels. Values are mean ± SE; * *p* < 0.05 vs. WKY; † *p* < 0.05 vs. PBS or NC; *n* = 6 per group in A; *n* = 4 per group in B and C.

Treatment with the miR155-5p mimic reduced superoxide anion production evidenced by the decreased DHE fluorescent intensity in VSMC of SHR (Figure 4A). Furthermore, the miR155-5p mimic inhibited NAD(P)H oxidase activity and NOX2 expression but not NOX4 expression in VSMC of SHR (Figure 4B,C). However, the miR155-5p mimic had no significant effects in VSMC of WKY rats (Figure 4A–C). It is known that oxidative stress greatly contributes to cell migration [27,28]. The antioxidant effect of the miR155-5p mimic might at least partially contribute to its inhibitory effect on the migration of VSMC from SHR.

### 3.4. Effects of miR155-5p Mimic on IL-1β and TNF-α Levels

**Figure 5:**
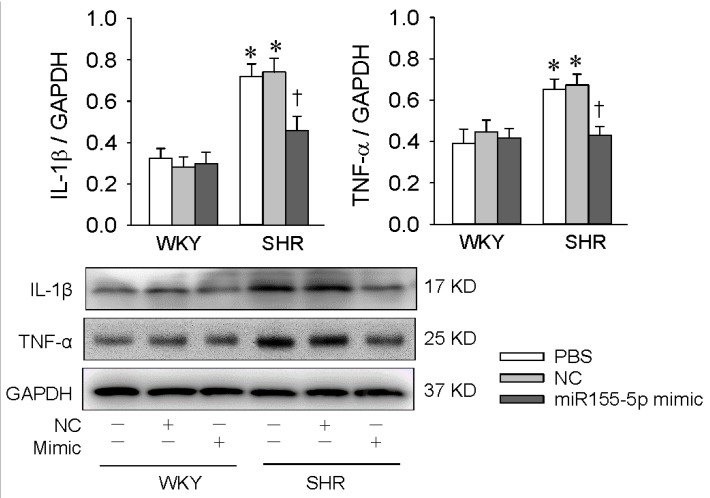
Effects of the miR155-5p mimic on IL-1β and TNF-α expression in VSMCs. VSMCs of WKY rats and SHR were treated with PBS, negative control (NC), or the miR155-5p mimic (50 nmol/L). Measurements were made 24 h after transfection. Values are mean ± SE; * *p* < 0.05 vs. WKY; † *p* < 0.05 vs. PBS or NC; *n* = 4 per group.

Chronic vascular inflammation is involved in the pathogeneses of hypertension, and oxidative stress promotes vascular inflammation and VSMC migration [8,29,30]. The miR155-5p mimic reduced IL-1β and TNF-α generation in VSMCs of SHR but not in those of WKY rats (Figure 5). The anti-inflammatory effects of miR155-5p might partially contribute to the attenuation of VSMC migration.

### 3.5. Effects of miR155-5p Overexpression on Ang II-Induced VSMC Migration

**Figure 6:**
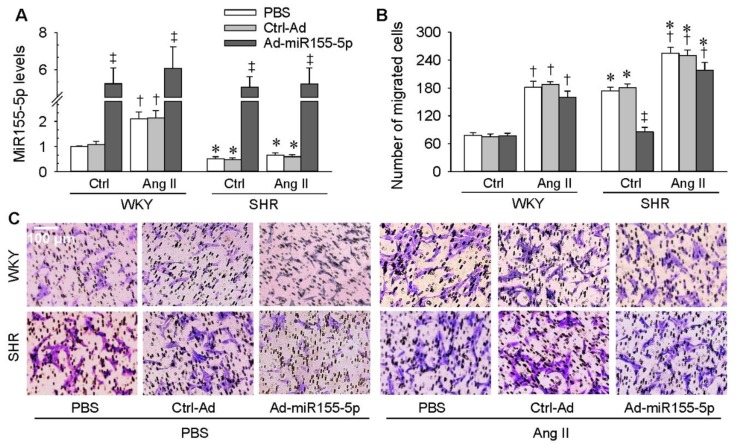
Effects of miR155-5p overexpression on angiotensin II (Ang II)-induced VSMC migration. VSMCs of WKY rats and SHR were treated with PBS, control adenovirus (Ctrl-Ad), or miR155-5p adenovirus (Ad-miR155-5p, 40 MOI) for 24 h, followed by PBS or Ang II treatment (100 nM) for another 24 h. (**A**) miR155-5p levels in VSMCs; (**B,C**) VSMC migration evaluated by the Boyden chamber assay. Values are mean ± SE; * *p* < 0.05 vs. WKY; † *p* < 0.05 vs. Ctrl; ‡ *p* < 0.05 vs. PBS or Ctrl-Ad; *n* = 6 per group.

We thought it would be interesting to ascertain whether miR155-5p had similar inhibiting effects on Ang II-induced VSMC migration, another model of VSMC migration. The Ang II model was selected according to the fact that Ang II plays crucial roles in the pathogenesis of hypertension and in vascular remodeling [31,32,33]. VSMCs of WKY rats and SHR were treated with PBS, Ctrl-Ad, or Ad-miR155-5p for 24 h, followed by PBS or Ang II treatment for another 24 h. The levels of miR155-5p were reduced in VSMCs of SHR compared with those of WKY rats. Exogenous Ang II increased miR155-5p expression in VSMCs of WKY rats, but not in those of SHR. Ad-miR155-5p treatment increased miR155-5p levels in all groups, confirming the effectiveness of miR155-5p overexpression (Figure 6A). Unexpectedly, miR155-5p overexpression only inhibited the migration of VSMC from SHR and had no significant effects on Ang II-induced migration of VSMC from WKY rats and SHR (Figure 6B,C).

### 3.6. Effects of miR155-5p Overexpression on Ang II-Induced Oxidative Stress and Inflammation

**Figure 7:**
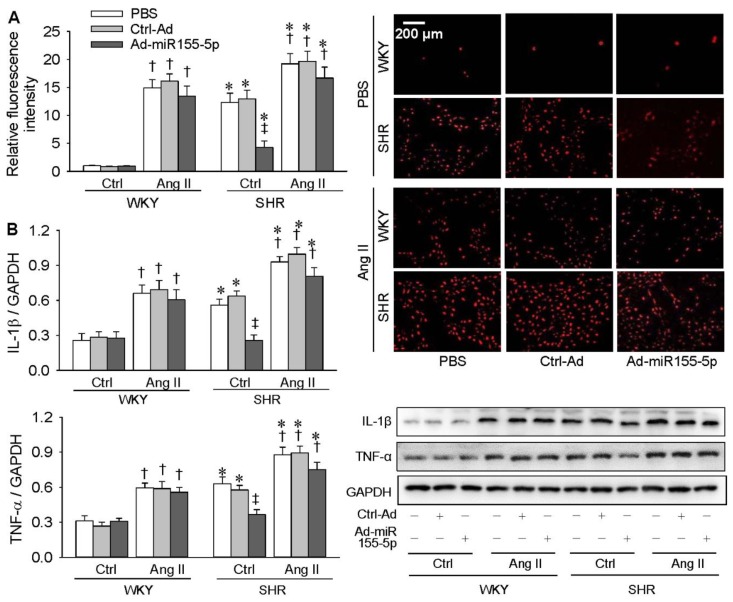
Effects of miR155-5p overexpression on Ang II-induced oxidative stress and inflammation in VSMCs. VSMCs of WKY rats and SHR were treated with PBS, control adenovirus (Ctrl-Ad), or miR155-5p adenovirus (Ad-miR155-5p, 40 MOI) for 24 h, followed by PBS or Ang II treatment (100 nM) for another 24 h. (**A**) DHE fluorescence staining for detecting ROS in VSMCs. (**B**) Interleukin-1β (IL-1β) and tumor necrosis factor α (TNF-α) protein expression levels in VSMCs. Values are mean ± SE; * *p* < 0.05 vs. WKY; † *p* < 0.05 vs. Ctrl; ‡ *p* < 0.05 vs. PBS or Ctrl-Ad; *n* = 6 per group in A; *n* = 4 per group in B.

MiR155-5p overexpression attenuated superoxide anion and IL-1β production in VSMCs of SHR but had no significant effects on Ang II-induced superoxide anion, IL-1β, and TNF-α production in the VSMCs of both WKY rats and SHR (Figure 7A,B). These results indicate that miR155-5p inhibits oxidative stress, inflammation, and cell migration in VSMCs of SHR but had no significant effects on exogenous Ang II-induced oxidative stress, inflammation, and cell migration in VSMCs of both types of rats.

## 4. Discussion

VSMC migration contributes to maladaptive vascular remodeling in hypertension and is associated with the pathogenesis of hypertension and related organ damage [34,35,36]. Our previous study showed that extracellular vesicles derived from aortic adventitial fibroblasts of normal rats inhibited VSMC proliferation by delivering miR155-5p to VSMCs [17]. The primary new findings of the present study are that miR155-5p inhibits the migration of VSMC from SHR by suppressing ACE expression and its downstream production of superoxide anion and inflammatory factors in VSMCs of SHR. However, miR155-5p had no significant effects on exogenous Ang II-induced VSMC migration and production of superoxide anion and inflammatory factors.

It is known that ACE converts Ang I into Ang II, which is critical in promoting VSMC migration via AT_1_ receptors [35], and is involved in vascular remodeling in hypertension [37,38,39]. The level of miR155-5p was reduced in VSMCs of SHR, and ACE was one of the targets of miR155-5p [17]. We found that the miR155-5p mimic inhibited the migration of VSMC from SHR, which was accompanied by the reduction of ACE mRNA and protein expression, while the miR155-5p inhibitor promoted the migration of VSMC of both WKY rats and SHR, which was accompanied by the upregulation of ACE mRNA and protein expression. These findings indicate that increased miR155-5p attenuates the migration of VSMC from SHR by inhibiting ACE expression. Endogenous miR155-5p still plays a role in inhibiting VSMC migration, although miR155-5p level is reduced in VSMCs of SHR. The findings were further confirmed by the evidence that miR155-5p overexpression abolished the enhanced migration of VSMC from SHR.

Chronic vascular oxidative stress and inflammation greatly contribute to the pathogeneses of hypertension and several vascular diseases [40,41,42]. It is known that ROS promote inflammation, and inflammation also enhances oxidative stress in cardiovascular diseases [43,44,45]. We found that the miR155-5p mimic attenuated oxidative stress and inflammation in VSMCs of SHR, as evidenced by reduced superoxide anion production, NAD(P)H oxidase activity, and expressions of NOX2, IL-1β, and TNF-α in VSMCs of SHR. The role of miR155-5p in attenuating oxidative stress and inflammation was further confirmed by the effects of miR155-5p overexpression in VSMCs of SHR. Ang II is a peptide that promotes VSMC proliferation, migration, oxidative stress, inflammation, and vascular remodeling [46]. It is known that Ang II activates NAD(P)H oxidases (NOXs) and thereby stimulates superoxide anion production and inflammation [24]. Based on the findings that miR155-5p inhibits ACE expression, we speculate that the roles of the miR155-5p mimic or miR155-5p overexpression in inhibiting VSMC migration might be mediated by the inhibition of ACE expression and thereby of ACE downstream signal pathways, including those leading to reduced Ang II production, NOX2 expression, oxidative stress, and inflammation in VSMCs of SHR. This speculation is highly supported by the findings that miR155-5p had no significant effects on exogenous Ang II-induced VSMC migration, oxidative stress, inflammation. Nevertheless, miR155-5p plays essential roles in inhibiting VSMC migration, oxidative stress, and inflammation by preventing ACE-dependent Ang II production in VSMCs of SHR.

ACE level was increased, while miR155-5p level was reduced in VSMCs of SHR compared to VSMCs of WKY rats; miR155-5p negatively regulated ACE expression. Although miR155-5p mimic reduced ACE expression in VSMCs of both WKY and SHR, it inhibited oxidative stress, inflammatory factors production, and cell migration only in VSMCs of SHR. A possible explanation is that the inhibitory effects of ACE downregulation caused by miR155-5p may be counteracted by some compensatory effects in normal rats, because adequate superoxide anion levels, inflammatory factor production, and cell migration are necessary to maintain normal VSMC structure and function. In SHR, reduced miR155-5p and increased ACE expression may become dominant factors, so miR155-5p reduces ACE expression and thereby attenuates oxidative stress, inflammatory factors production, and cell migration.

It is well known that Ang II greatly contributes to the pathogenesis of hypertension and to vascular remodeling [31,32,33]. Ang II is commonly used as an agent to induce experimental hypertension in animals or to induce VSMC proliferation, migration, oxidative stress, and inflammation in vitro [47,48,49]. It is noted that miR155-5p inhibited cell migration, oxidative stress, and inflammation in VSMCs of SHR, but not Ang II-induced VSMC migration, oxidative stress, and inflammation. A reasonable explanation is that miR155-5p inhibits ACE expression and, thereby, reduces endogenous Ang II production in VSMCs of SHR. In Ang II-treated VSMCs, although miR155-5p inhibits ACE expression and reduces endogenous Ang II production, a large amount of exogenous Ang II still promotes VSMC migration, oxidative stress, and inflammation.

ACE inhibitors are widely used as drugs for the treatment of hypertension. Although ACE inhibitors are relatively safe for short-term use, an increased risk of cancer may be associated with ACE inhibitors for long-term use [50]. We found that miR155-5p attenuated migration, oxidative stress, and inflammation in VSMCs of SHR. Furthermore, we recently showed that miR155-5p attenuated VSMC proliferation and vascular remodeling in SHR [17]. These findings suggest that miR155-5p might be a potential prospect used for inhibiting ACE expression and thereby for attenuating vascular remodeling in hypertension, which needs further investigation.

## 5. Conclusions

MiR155-5p inhibits cell migration in VSMCs of SHR by suppressing ACE expression, oxidative stress, and inflammation. However, miR155-5p had no effects on exogenous Ang II-induced VSMC migration, oxidative stress, and inflammation.

## Data Availability

Raw data for this study are available from the corresponding author on reasonable request.

## Supplementary Materials

The following are available online at https://www.mdpi.com/2076-3921/9/3/204/s1, Table S1: Primers for real-time quantitative PCR analysis in rats.
