# Supplementary material for: MiR155-5p Inhibits Cell Migration and Oxidative Stress in Vascular Smooth Muscle Cells of Spontaneously Hypertensive Rats
Source: Antioxidants (Basel). 2020 Mar 1;9(3):204. doi: 10.3390/antiox9030204 (PMC7140008; doi:10.3390/antiox9030204)
Supplement: Supplementary file 1 [file antioxidants-09-00204-s001.pdf]

Table S1 Primers for real-time quantitative PCR analysis in rats

Gene	Primer	Sequence
Rat ACE	Forward	5'- CAGCTTCATCATCCAGTTCC -3'
	Reverse	5'- CTAGGAAGAGCAGCACCCAC -3'
Rat GAPDH	Forward	5'- GGAAAGCTGTGGCGTGAT -3'
	Reverse	5'- AAGGTGGAAGAATGGGAGTT -3'
Rno-miR155-5p		5'-CGTTAATGCTAATTGTGATAGGGGT-3'
Rno-miRU6		5'- TTGGAACGATACAGAGAAGATTAGCAT-3'
